# Assessment of Shear Fracture Energy in Hybrid Composites with Natural and Synthetic Fibers

**DOI:** 10.3390/ma17153794

**Published:** 2024-08-01

**Authors:** Ranulfo Neto, Bernardo Mendes, Bernardo Borges, Carolina Moreira, Eduardo Sampaio, Mariana Banea

**Affiliations:** 1 Center of Technology and Application of Composites, Federal University of Rio de Janeiro, Macaé 27965-045, Brazil; ranulfoufrj@gmail.com (R.N.);; 2 CEFET/RJ—Federal Center of Technological Education in Rio de Janeiro, Rio de Janeiro 20271-204, Brazil; 3 Laboratory of Adhesion and Adhesives, State University of Rio de Janeiro, Nova Friburgo 28625-570, Brazil; 4 CICECO—Aveiro Institute of Materials, Department of Materials and Ceramic Engineering, University of Aveiro, 3810-193 Aveiro, Portugal

**Keywords:** hybrid composite joints, adhesive joints, mode II, shear fracture energy, ENF

## Abstract

Composite materials made with synthetic fibers are extensively employed across a diverse array of engineering structures. However, from an environmental point of view, synthetic fibers do not represent the best choice, since they are not renewable and are not biodegradable as natural fibers. This study investigates the application of adhesive joints with hybrid composites, which combine natural and synthetic fibers, as potential replacements for traditional composites made solely from synthetic fibers. The main focus is on assessing the mechanical performance of these hybrid composites through end-notched flexure (ENF) tests on adhesive joints. Four different configurations of substrates were used, two with only one type of fiber (natural or synthetic) and two hybrids. Digital image correlation (DIC) analysis was conducted to provide detailed insights into the changes in displacement fields for the different configurations tested. The results indicate that adhesive joints with hybrid composites exhibit superior shear fracture energy (*G_IIC_*) compared with the joints with purely synthetic fibers. This enhancement in fracture toughness, attributed to the synergistic effects of the natural and synthetic fibers, suggests that hybrid composites could be a viable alternative, offering potential benefits in terms of sustainability and cost without compromising mechanical performance.

## 1. Introduction

Fiber-reinforced polymer (FRP) composite materials have emerged as indispensable components across a number of industrial sectors, encompassing aircraft, automobiles and offshore structures [1]. Their widespread adoption stems from a combination of factors, notably their exceptional strength-to-weight ratios, enhanced fatigue resistance, and reduced maintenance costs due to improved corrosion resistance [2]. In this dynamic environment, natural fiber-reinforced composites (NFRCs) have emerged as a compelling alternative, poised to address the pressing need for cost reduction and environmental sustainability [3]. Compared with synthetic fiber, natural fibers offer several advantages, such as having lower density, posing no health risks, being available from natural and renewable sources, and being biodegradable and cheaper on a per unit volume basis [4,5]. In contrast, natural fibers usually do not achieve the same level of specific stiffness and strength as synthetic fibers and present high moisture absorption. Recent studies forecast significant growth in the commercialization of natural fibers due to the discovery of innovative applications and advancements in manufacturing technologies. This expansion is primarily driven by the increasing emphasis on sustainability, which has emerged as a crucial focus for the industry in recent years [6].

The application of NFRCs has gained significant attention, particularly in non-structural automotive components like door panels, instrument panels, and boot liners [7]. However, for structural applications, NFRCs are rarely used due to the cited limitations related to stiffness and strength, great susceptibility to moisture absorption, and variability in fiber quality. Considering the potential benefits of NFRCs especially in terms of sustainability, the questions that arise are (i) how to improve the strength of NFRCs and (ii) how to eliminate (or mitigate) the moisture absorption of NFRCs. The work of de Queiroz H. et al. [8] investigated the effect of two fiber reinforcement architectures (2D and 3D) on the single-lap joint efficiency of NFRCs. The results showed that the reinforced joints successfully reached similar failure loads compared with the synthetic composite joints. Another strategy is the hybridization of the composite, which combines the unique properties of both natural and synthetic reinforcement materials within a single matrix. In addition to improving the mechanical properties of the composite, hybridization may also attenuate moisture absorption once the synthetic fibers provide a barrier to humidity given their hydrophobic nature [9]. Several works have investigated the effect of hybridization on adhesive joints in single-lap joints (SLJs), including symmetric and asymmetric cases and different numbers of layers of synthetic fibers [10,11,12]. In general, the failure loads of the hybrid adherend joints increase by increasing the number of external synthetic layers, reaching values very close to those obtained in adhesive joints with adherends made of synthetic fibers only. However, there is a gap in the literature concerning adhesive joints with hybrid substrates, specifically regarding fracture testing.

The Finite Element Method (FEM) is pivotal in the design and analysis of adhesive joints, contributing significantly to understanding and predicting the mechanical behavior and failure mechanisms of these systems. In this context, the cohesive zone models (CZMs) stand out as a powerful tool, combining a stress-based analysis and fracture mechanics to model damage onset and crack propagation [13,14]. The critical fracture energies in modes I and II (*G_IC_* and *G_IIC_*, respectively) are key properties used in the CZMs. To obtain *G_IC_*, the double cantilever beam (DCB) test is the most widespread, while for calculating *G_IIC_*, the end-notched flexure (ENF) test is the most used due to its simplicity and easy test setup [15].

The fracture characterization of NFRCs and their hybrid combinations can be studied solely in the laminate form (interlaminar fracture toughness) or by using the laminate as substrate in adhesive joints. Several works have been reported regarding the interlaminar fracture toughness of fiber-reinforced composites, whether for loads in mode I [16,17,18] or mode II [17,19,20,21,22]. In general, increasing the number of layers increases stiffness and interlaminar fracture toughness [16]. Another way to improve the fracture toughness is by adding nanoparticles of TiO_2_ [17]. Zhang et al. [18] investigated the behavior of hybrid joints made of flax and glass fibers submitted to loads in mode I. The hybrid configuration had a higher value of interlaminar shear strength than the pure configurations of flax and glass fibers. Regarding the interlaminar fracture toughness in mode I, similar values for the hybrid and pure flax laminates were obtained, while the glass laminates had the lowest value. Several factors may affect the values of interlaminar fracture toughness, such as the layup configurations [19], fiber architectures [20], and curing temperature [21]. While several studies have investigated interlaminar fracture toughness, to the best of the authors’ knowledge, there are no studies in the literature where fracture energies are evaluated in adhesive joints with substrates made of natural fibers or hybrid natural/synthetic composites.

This work aimed to investigate experimentally the behavior of adhesive joints by using ENF samples of natural fibers, synthetic fibers, and two configurations of interlaminar hybrid fiber-reinforced polymer composites. The influence of hybridization on both maximum load and shear fracture energy were evaluated. The digital image correlation (DIC) technique was used in order to compare the displacement fields of all configurations tested. Furthermore, a failure mechanism analysis was carried out for the samples of all configurations. As a most impactful result, the values of *G_IIC_* for the adhesive joints with hybrid configurations were higher than the joints with purely synthetic fibers.

## 2. Materials and Methods

### 2.1. Materials

Bidirectional jute (T4, model N 30 002 N1, supplied by Sisalsul, São Paulo, Brazil) and unidirectional glass fiber wovens (supplied by Texiglass, São Paulo, Brazil) were used as reinforcements for the substrates. Jute is one of the most widely available and cost-effective natural fibers, which justifies its application in this work. Glass fibers are suitable for a wide range of applications, presenting costing advantages in relation to carbon fibers, making them more economically viable for large-scale applications. A two-component epoxy resin (PIPEFIX 50/90/100) (Fine Composites, Nova Friburgo, Brazil) was used to manufacture the laminates. The bi-component structural NVT 201E epoxy adhesive (Fine Composites, Nova Friburgo, Brazil) was used for joining the composite substrates. It is a brittle adhesive with weight ratio of 100:52 (resin to hardener), glass transition temperature of 71 °C, elastic modulus of 3.13 Gpa, and tensile strength of 28.7 MPa [13]. The curing time for both manufacturing processes (substrate and adhesive joint) is 24 h, as recommended by the suppliers.

### 2.2. Joint Geometry and Manufacturing

**Figure 1:**
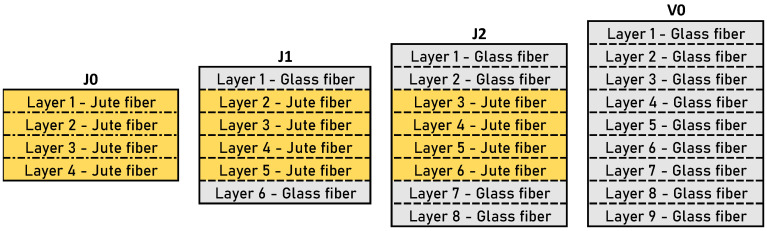
Stacking sequence of natural, hybrid, and synthetic substrates.

Four distinct configurations were fabricated and tested: (i) a laminated structure with four layers of reinforcement, all utilizing jute fibers (J0); (ii) a laminated structure consisting of six layers, with the first and last layers being reinforced with unidirectional fiberglass laminae and the four inner layers being reinforced with jute fibers (J1); (iii) a laminated structure comprising eight layers of reinforcement, with unidirectional fiberglass laminae being used for the two outermost layers and the four inner layers being reinforced with jute fibers (J2); and (iv) a laminated structure with nine layers of reinforcement, all reinforced with unidirectional fiberglass laminae (V0). The numbers of layers for configurations J0 and V0 were not the same because the thickness of each lamina was very different. This way, the laminates did not present substantial differences in thickness. Figure 1 schematically represents the stacking sequence of all configurations. There was no variation in the stacking sequence of the unidirectional laminae (they were all positioned at 0°), although it has been reported that this factor influences shear fracture energy [23]. Two main experiments were performed for the four groups: (i) the laminates (not joints) were tested in tensile tests, and (ii) the adhesive joints were tested in three-point bending test (ENF).

The composite substrates (natural, hybrid, and synthetic) were manufactured by using the hand layup process at room temperature. Fibers and resin were prepared in controlled environments to maintain consistent quality and proportions. The laminae were stacked on top of each other with intermediary PIPEFIX lamination resin between the layers. In the course of manufacturing, each ply (jute or glass) was compressed with a metal roller to remove bubbles, maintain uniform thickness, and ensure homogeneity. Similar methodologies were used elsewhere [23,24,25]. Continuous visual inspection was conducted during the layup process to identify and correct any inconsistencies or defects immediately. Aiming to aid air and volatile release from the laminate, nylon-based peel ply layers were applied to both the top and the bottom surfaces of the substrates during layup for all configurations. Finally, after the curing process, the laminates were cut in the desired dimensions: (i) tensile tests, 220 mm × 15 mm, and (ii) ENF tests, 160 mm × 20 mm. The thicknesses of the laminates had the following values: J0—4.5 mm; J1—5.0 mm; J2—5.8 mm; and V0—3.8 mm. Tensile tests were performed for all groups to confirm consistency and quality, as detailed in Section 2.3.

**Figure 2:**
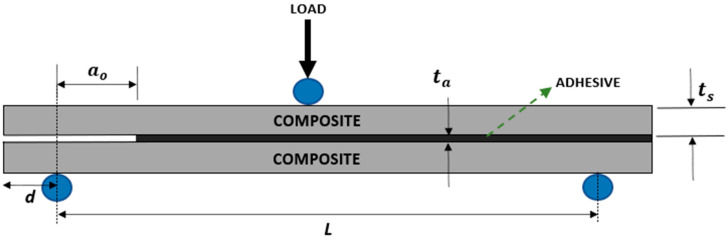
Schematic representation of specimens and boundary conditions for ENF test.

The substrates were joined by using epoxy (NVT 201E) in a mold, with pins installed to prevent substrate misalignment. Metal strips were positioned at the ends of the ENF joint to ensure constant adhesive thickness. Aiming to induce cohesive failures in the adhesive layer, a double bevel was made on the part of the metal strip in contact with the adhesive. These metal strips were removed after the curing process. The adhesive was applied on both laminates after the removal of the peel ply sheets. Then, the substrates were joined, and the excess adhesive was removed by using a spatula. The curing time was 24 h, as recommended by the suppliers. The schematic representation of the ENF specimens can be seen in Figure 2, where *L* = 130 mm, *d* = 15 mm, *a_o_* = 30 mm, *t_a_* = 0.3 mm, and *t_s_* had the values according to the thicknesses of the laminates (described in the previous paragraph). The width was 20 mm. Similar dimensions were used elsewhere [23].

### 2.3. Testing Conditions

At first, tensile tests were performed at room temperature in the composites (natural, hybrid, and synthetic) with a universal testing machine, Shimadzu model Autography AG-X Plus 100 kN (Kyoto, Japan), at a crosshead speed of 1 mm/min. Then, the adhesive joints were submitted to the ENF test at room temperature and under displacement control (0.5 mm/min). At least four specimens were tested for each configuration. For both tests (tensile and ENF), the load vs. displacement curves were recorded during the test. For the tensile test, these data (load vs. displacement) are enough to obtain both Young’s modulus and tensile strength, while for the ENF test, these data are needed for implementing the compliance-based beam method (CBBM), which was used to determine *G_IIC_* and is explained in Section 3. The boundary conditions of the ENF test are presented in Figure 2.

The two-dimensional digital image correlation (DIC) technique was used to compare the displacement fields of all joints tested. The images during the tests were captured every 2 s by using a high-resolution camera (BFS-U3-32S4M-C USB 3.1 Blackfly from FLIR with video lens cfzoom 13–130 mm, Hallandale Beach, FL, USA). The DIC program has an accuracy of 0.02 pixels.

Finally, the failure modes of the adhesive joints were visually analyzed and compared after the ENF tests.

### 2.4. Data Reduction Method

The compliance-based beam method (CBBM) is often used for analyzing the data from fracture tests in adhesive joints. Monitoring the progression of crack length during the fracture test is not an easy task, especially in the three-point bending test, since the crack tends to close due to the applied load, which impedes a clear visualization of its tip [26,27]. CBBM application addresses this issue, given that it depends only on the samples’ compliance during the test; this way, the direct measure of the crack length is not necessary [28]. Furthermore, this method also takes into consideration the fracture process zone (FPZ) ahead of the crack tip. The experimental compliance (*C*) can be written as a function of the crack length (*a*) by using Timoshenko beam theory, as presented in Equation (1).

(1) $$C=\frac{\left(3a^{3}+2L^{3}\right)}{8E_{1}B{t_{s}}^{3}}+\frac{3L}{10G_{13}Bt_{s}}$$

where *E*_1_ is the longitudinal Young’s modulus of the substrate, *G*_13_ is the transverse shear modulus of the substrate, and *B* is the width. The dimensions *L* and *t_s_* are presented in Figure 2.

The flexural modulus (*E_f_*) of each sample plays a crucial role in the load vs. displacement curve [28]. It can be obtained by rearranging Equation (1) and making *C* = *C*_0_ (initial compliance), *a = a_o_* (initial crack length), and *E = E_f_*; thus, Equation (2) is obtained.

(2) $$E_{f}=\frac{3a_{o}^{3}+2L^{3}}{8B{t_{s}}^{3}C_{0corr}}$$

where *C*_0*corr*_ is given by

(3) $$C_{0corr}=C_{0}-\frac{3L}{10G_{13}Bt_{s}}$$

By substituting in Equation (1) *E*_1_ with *E_f_* and *a* with *a_e_* (equivalent crack length), Equation (4) can be written.

(4) $$a_{e}=[\frac{C_{corr}}{C_{0corr}}a_{o}^{3}+\frac{2}{3}(\frac{C_{corr}}{C_{0corr}}-1)L^{3}{]}^{\left(1/3\right)}$$

*C_corr_* can be obtained by using Equation (3) and substituting *C*_0_ with *C*.

Finally, the critical fracture energy in mode II (*G_IIc_*) is then obtained by using the Irwin–Kies relation [29] as follows:

(5) $$G_{IIc}=\frac{P²}{2B}\frac{dC}{da}=\frac{9P^{2}{a_{e}}^{2}}{16B^{2}{t_{s}}^{3}E_{f}}$$

where *P* is the load applied by the universal testing machine.

The CBBM method was applied to all samples of all stacking sequence configurations (J0, J1, J2, and V0) studied in this work.

## 3. Results and Discussion

### 3.1. Tensile Tests

**Figure 3:**
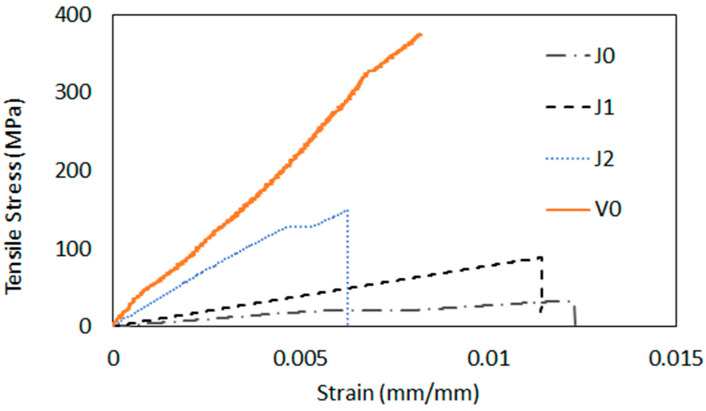
Representative tensile stress–strain curves of the composite samples for all groups tested.

The tensile tests conducted on the composites with different configurations revealed significant variations in mechanical properties among the samples (see Figure 3). As expected, the composite with jute fiber (J0) exhibited the lowest tensile strength, with an average of 37.45 MPa. In contrast, the J1 hybrid configuration showed a substantial increase of approx. 162% in tensile strength, while from the J1 configuration to the J2 configuration, the average value increased by approx. 53%, varying from 98.18 MPa to 149.87 MPa (see Table 1). The glass fiber composite (V0) demonstrated the highest tensile strength, with an average value of 357.47 MPa. The same tendency was observed for Young’s modulus, as seen in Table 1. These behaviors were expected, since glass fibers have higher intrinsic strength and stiffness compared to jute fibers, which typically consist of lignocellulosic materials that are less strong and more prone to deformation [30].

### 3.2. ENF Tests

#### 3.2.1. Maximum Load

**Figure 4:**
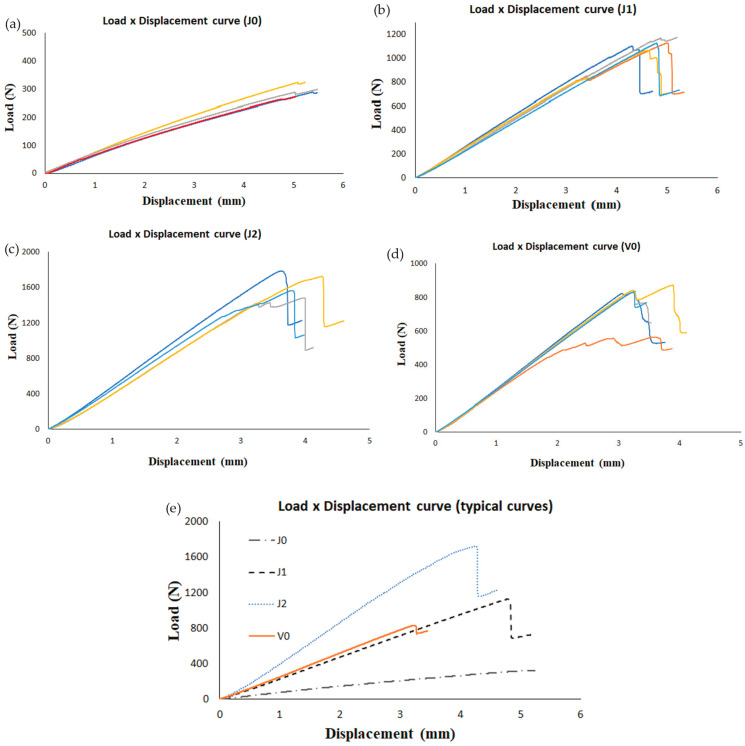
Load–displacement curves of ENF joints as a function of the material: (**a**) J0, (**b**) J1, (**c**) J2, (**d**) V0, and (**e**) typical curves of each configuration.

The load vs. displacement curves for all samples tested at each configuration are presented in Figure 4a–d. For the joints from the J0 group, no sudden drops in load were observed, but small drops were recorded. It was not possible to see when the crack had fully propagated, as often occurs in ENF tests, but it looks like this occurred right after the load dropped gently, since it is the expected behavior for a brittle adhesive. For the other groups (J1, J2, and V0), the load dropped suddenly for some samples, and small drops were observed in other cases, which indicates slightly smoother failure progression. Substantial increases in maximum loads were observed for both the J1 and J2 groups (see Table 2). The insertion of only two layers of fiber glass (one at each end—J1) is enough to increase the maximum load in 236% in relation to J0. A further increase is achieved by adding one more layer at each end (J2). These increases occurred due to the way stress is distributed during the ENF test. The top layer (where the punch makes contact) undergoes maximum compression, while the bottom layer experiences the highest tensile stress, which decreases linearly towards the middle of the joint. Thus, in adhesive joints with hybrid composite substrates, outer synthetic fibers enhance resistance to bending loads and improve overall performance. Comparing J1 and J2 groups, the latter had a maximum load 44% higher. Smaller increases were reported in single-lap joints (SLJs) considering a similar comparison between hybrid joints [10], but the authors used carbon fibers instead of glass fibers as reinforcement. For the V0 group, small drops were observed in most samples (see Figure 4d), similar to what has been reported elsewhere [23]. Although the V0 group had a greater maximum load than J0, it was smaller than that for the J1 group (see Table 2), which was not expected and was not reported in similar works in SLJs [10]. The stiffness for both the V0 and J1 joints was similar, but the maximum load of J1 was 44% higher than that of V0. The joints from the J1 group had one more interface than V0, since for this case, there was a contact between natural and synthetic fibers. This interface had a very relevant effect, improving the interfacial bonding between the fibers and the matrix and contributing to the increase in maximum load. Figure 4d presents the typical curves for each configuration. The stiffness increased from J0 to J1 and from J1 to J2, as expected. As for V0, the stiffness was similar to that obtained for the J1 group, as already reported.

#### 3.2.2. Fracture Energy

**Figure 5:**
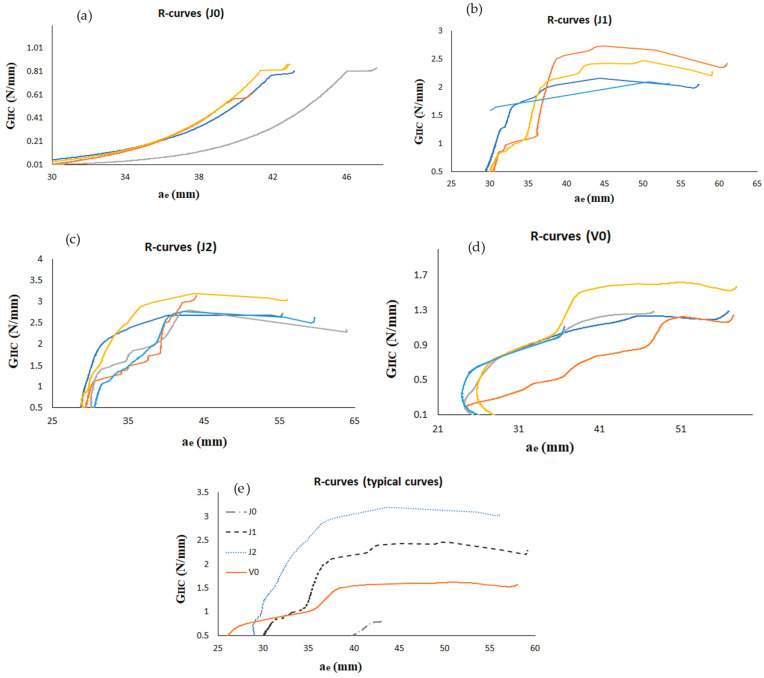
Resistance curves: (**a**) J0, (**b**) J1, (**c**) J2, (**d**) V0, and (**e**) typical curves of each configuration.

Figure 5a–d show all the resistance curves (R-curves) obtained for each group analyzed (for all specimens), and a comparison of typical curves is presented in Figure 5e. Table 3 summarizes the data obtained in the experiments. When only jute fibers were used in the substrates (J0 group), the lowest value of *G_IIC_* was obtained. It was expected, since jute adherends had the lowest mechanical properties considering the groups analyzed in this work, as presented in Section 3.1. Still, for the V0 group, a clear plateau representing *G_IIC_* was formed for all samples; however, it was seen only over a small range of the crack length. For the other groups (J1, J2, and V0), the R-curves also presented clear plateaus (with little oscillations in some cases), but now, they were distributed in longer ranges of crack length. *G_IIC_* had a substantial increase of 147% in the J1 group, due to the enhancement in the mechanical properties achieved by the addition of one layer of fiber glass at each end of the ENF joint. The fracture energy continued to grow for the J2 group (35% higher than for J1), since now the properties were even more improved. Now, for the V0 group, *G_IIC_* was 53% higher than for the J0 group; however, it was smaller than the values obtained for the J1 and J2 groups. It is clear that the additional interfaces between glass and jute layers presented in the J1 and J2 groups were very strong and thus contributed to the increase in fracture energy. The morphology of the unidirectional fiberglass is superficial and uniform (since they are formed by smooth tubes) [31], while for jute fibers, the morphology is more random; thus, the friction force can be greater, resulting in higher absorption of energy in the substrate (especially at the interface between jute and glass), thus increasing *G_IIC_* in the J1 and J2 groups compared with V0. It demonstrates the relevance of investigating the adhesive joints’ behavior, where the interface plays a crucial role. As reported in Section 3.1, the mechanical properties increased in this sequence: J0, J1, J2, and V0; but now, in the ENF tests, both the maximum load and fracture energy increased in another sequence: J0, V0, J1 and J2. It should be noted that only tensile loads were applied in the tensile tests (Section 3.1), while for the ENF tests, the loads were purely shear.

#### 3.2.3. DIC Analysis

**Figure 6:**
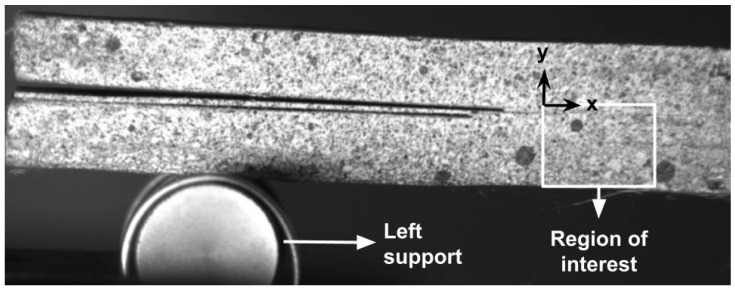
Region of interest used for digital image correlation (DIC).

The region of interest used in the DIC analysis is shown in Figure 6. The horizontal part measured 4.8 mm, while the vertical part was 3.7 mm long. The region included the adhesive and some parts of the superior and inferior substrates. Due to the great displacements caused by the ENF test, the region of interest needed to include a considerable part of the inferior substrate. The images used for the analysis were divided into 21 elements along both the x- and y-axes. The search sub-images were set to be twice the size of reference sub-images. For the calibration from pixels to millimeters, the thickness of each specimen was considered.

**Figure 7:**
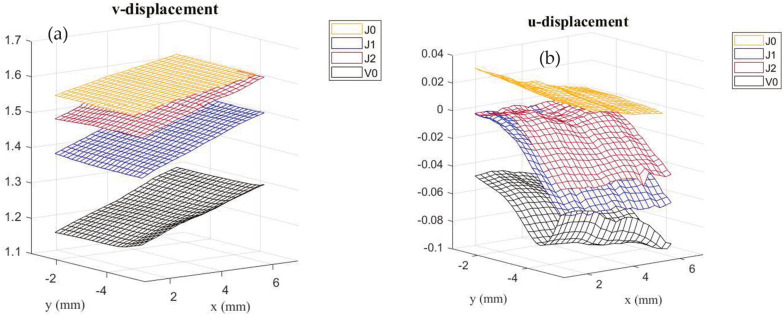
Vertical (**a**) and horizontal (**b**) displacement fields of all joints’ configurations.

A constant machine displacement of 2 mm was considered in this analysis to compare the displacement fields of all the joints’ configurations. Figure 7 represents the vertical (a) and horizontal (b) displacement fields for the natural, hybrid, and synthetic joints. The vertical axis represents the displacement in mm, while x and y are those dimensions from the region of interest in Figure 6. Figure 7a indicates that by increasing the stiffness of the substrates the vertical displacement decreased considerably. The J0 joints (pure jute) had the highest values of vertical displacements, while the V0 joints (pure glass fibers) had the lowest values. As expected, the hybrid configurations (J1 and J2) had intermediate behaviors. It is clear that the stiffness of the substrates influences the values of fracture energy; however, higher stiffness does not necessarily imply higher values of *G_IIC_*. The horizontal displacement field also plays an important role. According to Figure 7b, the V0 group had the highest values of horizontal displacements, while the J0 group had the lowest values. Interestingly, the order of increase in horizontal displacement was the opposite of that observed for vertical displacement. This may indicate that considering the same machine displacement, the crack propagation may have occurred first for the V0 configuration, followed by J1 and J2, which had similar horizontal displacement fields. Consequently, hybridization acted by delaying crack propagation and leading the J1 and J2 configurations to absorb more energy during the test, resulting in higher values of fracture energies. The J0 group had the lowest values of horizontal displacements; however, this did not imply higher values of fracture energy, since the predominant failure for this group occurred in the substrate, as discussed in the next section.

#### 3.2.4. Failure Modes

**Figure 8:**
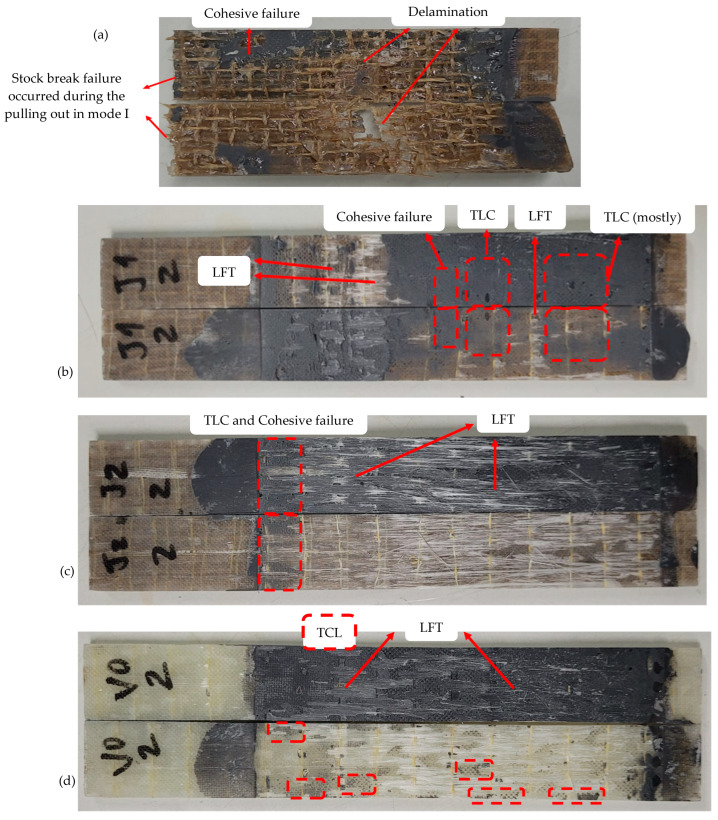
Representative failure modes at the tested joints: (**a**) J0, (**b**) J1, (**c**) J2, and (**d**) V0.

After the ENF tests, the substrates were manually separated to verify the joints’ failure modes. The representative fracture surfaces from each group are presented in Figure 8 via macro-scale pictures. As expected in adhesive joints with composite substrates, mixed failure modes occurred for all groups [12]. It was not possible to obtain the entire fracture surface in the J0 specimens because brittle failures occurred in the substrates during the pulling out in mode I; this way, the specimen was broken in some parts. The largest piece of one of the J0 specimen is presented in Figure 8a, where small regions of cohesive failures, as well as more severe and widespread fiber removal and bundle failures, which is called delamination, can be observed. There are no data available in the literature to compare with the obtained results, since fracture tests using vegetal fibers have been not reported so far. For the J1 sample, cohesive failure and thin layer cohesive (TLC) failures were predominant, as can be seen in Figure 8b. TCL failure occurs when a thick layer of adhesive stays on one adherend and a very thin adhesive layer (adhesive dusting) stays on the other. It explains the much larger value of *G_IIC_* obtained for J1 compared with J0. Light fiber tear (LFT), which is characterized by the presence of a thick adhesive layer on one side and very little adhesive on the other side (or absence), with some surface resin and few fibers removed from the interface, was also observed in small parts of the J1 sample. The LFT failure mode is expected to be predominant in joints with glass fiber substrates [12]. It occurred not only in the V0 samples but also in the J2 samples, where four layers of glass fibers were applied (two at each end), as can be visualized in Figure 8c,d. In the J2 group, TLC and cohesive failures were also observed in small regions, but LFT was clearly predominant. While the J1 samples had two different interfaces (jute/glass and glass/adhesive), the J2 samples had an additional interface, glass/glass, which resulted in different failure modes and *G_IIC_* values (in J2, it was 35% higher than in J1). For the V0 samples, there were small regions of TCL failure mode, but LFT was predominant, as already reported. In general, the J0 group had higher final displacements than V0, J1, and J2 (as shown in Section 3.2.1), which contributed to the bending of the specimen and induced tensile stress. This may explain the difference in terms of failure between J0 and the other groups. It should be noted that the different failure modes observed for the different configurations occurred for various reasons, primarily due to the different interface forces, but not due to the manufacturing process, since it was standardized for all configurations (as detailed in Section 2.2).

## 4. Conclusions

The effect of hybridization of adhesive joints with natural and synthetic fibers submitted to shear loads was experimentally investigated. The tensile tests confirmed the expected tendency, where the highest tensile strength and Young’s modulus were obtained for the groups of joints with higher stiffness (V0), followed by hybrid configurations (J2 and J1) and finally the joints with solely jute fibers (J0). For the ENF tests, the results of the hybrid configurations (J1 and J2) were very promising, since higher maximum loads were obtained compared with the joints with synthetic substrates (V0). J0 had the smallest value, as expected. In the same direction, the J0 joint had the smallest value of *G_IIC_*. However, the values of *G_IIC_* were higher for the hybrid configurations (J1 and J2) than for the V0 configuration. This behavior was attributed to the additional interface present in the hybrid configurations, located between the glass and jute fibers. The different morphologies of the unidirectional fiberglass (superficial and uniform) and jute fibers (random) generate higher friction forces at the interface between jute and glass, resulting in higher energy absorption in the substrate.

The DIC analysis quantified the different field displacements in both the horizontal and vertical directions, while the failure mode investigation showed that the addition of synthetic fibers alters significantly the type of failure, avoiding delamination in the substrates.

In terms of the hand layup process, even if the process is carried out with considerable attention to detail by a skilled professional, it is a manual method that inherently involves some degree of variability. The hand layup process is known to present inevitable variations due to factors such as variations in the application of resin and the alignment of the fibers, variability in the handling and mixing of the composite materials, and fluctuations in ambient conditions during the layup process. Since these factors may influence the experimental results, other manufacturing processes may be used in future research, such as vacuum bagging and resin transfer molding.

Further investigations are needed to investigate the mode I and mixed-mode effects of hybridization. Furthermore, additional studies are necessary to investigate the effect of moisture absorption in hybrid adhesive joints with natural and synthetic substrates. For mode I, DCB tests can be performed with the same four configurations used in the current work, aiming to investigate the effect of hybridization on fracture energy in mode I (*G_IC_*). Similarly, mixed-mode tests need to be performed, but with different ratios between *G_I_* and *G_II_*. For the investigation of moisture absorption, ENF tests must be performed in saturated samples for the four configurations presented, and the values of fracture energy must be compared with those obtained in the samples without saturation.

## Figures and Tables

**Table 1:** Young’s modulus and tensile strength of all groups tested.

Group	Young’s Modulus (GPa)	Tensile Strength (MPa)
J0	4.18 ± 0.34	37.45 ± 5.10
J1	7.9 ± 0.64	98.18 ± 8.52
J2	10.88 ± 1.69	149.87 ± 6.34
V0	38.9 ± 2.47	357.47 ± 18.99

**Table 2:** Maximum loads and standard deviations for all groups.

Group	Fmax (N)	% Increase in Relation to J0
J0	337.31 ± 51.18	-
J1	1131.93 ± 40.50	236%
J2	1626.36 ± 102.99	382%
V0	786.37 ± 88.77	133%

**Table 3:** Critical fracture energies and standard deviations for all groups.

Group	*G_IIc_* (N/mm)	% Increase in Relation to J0
J0	0.87 ± 0.15	-
J1	2.15 ± 0.33	147%
J2	2.90 ± 0.38	233%
V0	1.33 ± 0.14	53%

## Data Availability

The original contributions presented in the study are included in the article, further inquiries can be directed to the corresponding author.
